# Removal of overextended root canal filling beyond the apex in lower premolar teeth using two different retreatment rotary files versus manual files: a comparative in vitro study

**DOI:** 10.1186/s12903-026-09571-1

**Published:** 2026-08-20

**Authors:** Khadijah Ahmed Sadek, Sybel Mokhtar Mahmoud Moussa, Ahmed Mostafa Mobarak

**Affiliations:** Conservative Dentistry Department, Faculty of Dentistry, Alexandria University, Champolion St., Alexandria, 21527 Egypt

**Keywords:** Retreatment, Overextended, Root canal filling, Mandibular premolars, XP-endo Finisher R

## Abstract

**Background:**

The aim of the study was to compare the time required for complete removal of overextended root canal filling in lower premolar teeth using two different rotary file systems; ProTaper Universal Retreatment (PTUR) system, XP-endo Retreatment (XPER) system versus manual Hedstrom (H-Files).

**Methods:**

Thirty-six extracted permanent mandibular premolars with single canal were set to a standard length of 19 mm and prepared beyond the apical foramen by 3 mm up to F2 file of the ProTaper Gold system. Overextended obturation was performed by a size (25/08) gutta-percha and AH-Plus sealer using cold lateral condensation technique. Teeth were left for 7 days to ensure complete setting of sealer, then divided into 3 groups (*n* = 12) according to the gutta-percha removal method (Group Ι: H-Files, Group ΙΙ: PTUR, Group ΙΙΙ: XPER (XP-endo Shaper (XPES) + XP-endo Finisher R (XPEF-R)). Time taken was recorded and statistically analyzed.

**Results:**

The XPER technique showed a statistically significant reduction in the time taken to remove the overextended gutta-percha compared to H-files and PTUR groups (*P* < .05), with the XPER group showing the shortest time, followed by PTUR, and then H-files.

**Conclusions:**

XPER technique was significantly faster in removing the experimentally overextended gutta-percha than H-files and PTUR files, demonstrating superior procedural time efficiency.

## Introduction

Overextension during root canal treatment may occur due to inaccurate working length determination, instrumentation beyond the apical foramen, inflammatory apical root resorption, or an underdeveloped root apex [1]. Obturation filling materials should be confined to the root canal system, as extrusion beyond the apical foramen may induce a periapical inflammatory response and contribute to necrosis of the cementum, periodontal ligament, and surrounding bone [2]. In addition, overinstrumented and overfilled teeth have been associated with higher rates of treatment failure [3–5].

Non-surgical retreatment is considered the preferred initial management for endodontically treated teeth with persistent apical periodontitis. This approach involves removal of the existing root canal obturation material, re-establishment of apical patency, thorough chemomechanical debridement and reshaping of the root canal system, followed by re-obturation to prevent reinfection [6].

Hand, rotary, and reciprocating file systems are used during retreatments. D1, D2, and D3 files are the constituents of the ProTaper Universal Retreatment (PTUR) system. D1 files have an active cutting tip that facilitates initial access, whereas D2 and D3 have inactive tips to minimize methodological errors during retreatment [7].

Recently, XP-endo Retreatment file system (XPER) (FKG Dentaire, Switzerland) was manufactured; it consists of DR1 (from D-Race system), XP-endo Shaper (XPES), and XP-endo Finisher R (XPEF-R) [8]. This file system is made from MaxWire (Martensite-Austenite Electropolishing-Flex, FKG), a thermomechanically treated nickel-titanium alloy [7] that transforms from martensite to austenite at temperatures of 35 °C or higher. This gives the file a semi-circular shape of the file, allowing it to perform an eccentric rotational motion [9]. The DR1 file features an active tip designed to facilitate navigation of the coronal third of the canal [8]. The XPES (30/0.04) has a snake-like configuration with a triangular cross-section and operates as a single-file system in continuous rotary motion [10]. The XPEF-R instrument is an advancement of the XP-endo Finisher (XPEF). It is characterized by a larger core diameter (size 30) than the original XPEF (size 25) and was specifically developed for retreatment procedures. In the austenitic phase, the instrument assumes a spoon-shaped configuration, maximizing contact with the canal walls and enhancing the removal of filling materials. This zero-taper finishing instrument effectively removes residual filling material from oval-shaped canals, demonstrating outcomes comparable to those achieved with the XPEF during primary treatment [8]. To adapt to canal morphology, the instrument expands to a diameter of up to 6 mm, approximately 100 times greater than a conventional instrument of the same nominal dimensions [8, 11].

Although prevention of apical overextension remains the primary clinical objective, clinicians may encounter previously treated teeth with overextended filling beyond the apex requiring nonsurgical retreatment. In such cases, removal of overextended material represents an additional procedural challenge. Investigating removal techniques should therefore be viewed as management of an existing complication rather than acceptance of overfilling as a treatment outcome. According to the current literature, no study has investigated the time required for the XPER system to completely remove overextended filling material. Previous research has predominantly focused on evaluating the efficacy of XPER systems for the removal of root canal filling materials during conventional retreatment procedures. In contrast, the present study specifically focused on the removal of gutta-percha overextended 3 mm beyond the apex, an area that has been insufficiently addressed in the existing literature. Therefore, the objective of this study was to compare the time required by H-files, PTUR, and XPER systems to remove experimentally standardized overextended root canal filling material from mandibular premolars with a single root canal.

The null hypothesis proposed that the file systems being tested would not differ significantly.

## Materials and methods

### Specimen selection and preparation

The Research Ethics Committee at the Faculty of Dentistry, Alexandria University (00010556/2024) approved the research protocol. Sample size determination was performed using G*Power (Version 3.1.9.7). The sample size was decided based on a prior study [12], supposing 80% power and a 5% alpha error. The requisite minimum sample size per group was 11 teeth, raised to 12 to account for laboratory processing errors. The total sample size required was 3 × 12 = 36 teeth.

Consequently, thirty-six human mandibular premolars were freshly extracted for periodontal or orthodontic reasons with patient consent. Only teeth with single roots and single canals, with curvature less than 5° according to Schneider were used in this study [13]. Following cleaning under a dental operating microscope, teeth were kept in a saline solution containing phosphate for stabilization (Biowest, USA) until needed. To ensure standardization, the teeth were flattened at the cusps until they measured 19 mm using a diamond TR bur with a high-speed handpiece (W&H Dentalwerk, Bürmoos, Austria).

Apical patency was confirmed using a #10 k-file after access cavity preparation, and a #15 k-file was used to record the working length. To establish a standardized experimental model of pronounced apical overextension, the instrumentation length was extended 3 mm beyond the apical foramen. This extension was created solely for experimental standardization and does not represent a clinically acceptable working length. The root canals were instrumented using the ProTaper Gold file system till the F2 file (25/08) (Dentsply Sirona, Switzerland), as advised by the manufacturer.

Across instrumentation, 2 ml of 5.25% sodium hypochlorite (NaOCl) (Chloraxid 5.25%, Cerkamed, Poland) was used after each file, then a 3 ml of 17% EDTA (PD-EDTA 17%, Switzerland) followed by saline as a final flush.

Subsequently, obturation was performed using the cold lateral compaction technique with size (25/08) gutta-percha cones (Dentsply Sirona, USA), AH-Plus sealer (Dentsply DeTrey, Konstanz, Germany) and accessory cones. The obturation quality was assessed radiographically. To allow the sealer to fully set, the specimens were maintained at 37 °C with 100% humidity for 7 days [14]. To control potential confounding factors, a single operator performed all procedures to avoid operator-dependent variability, and all experiments were conducted in a cabinet set at 37 °C [12].

### Retreatment

**Fig. 1:**
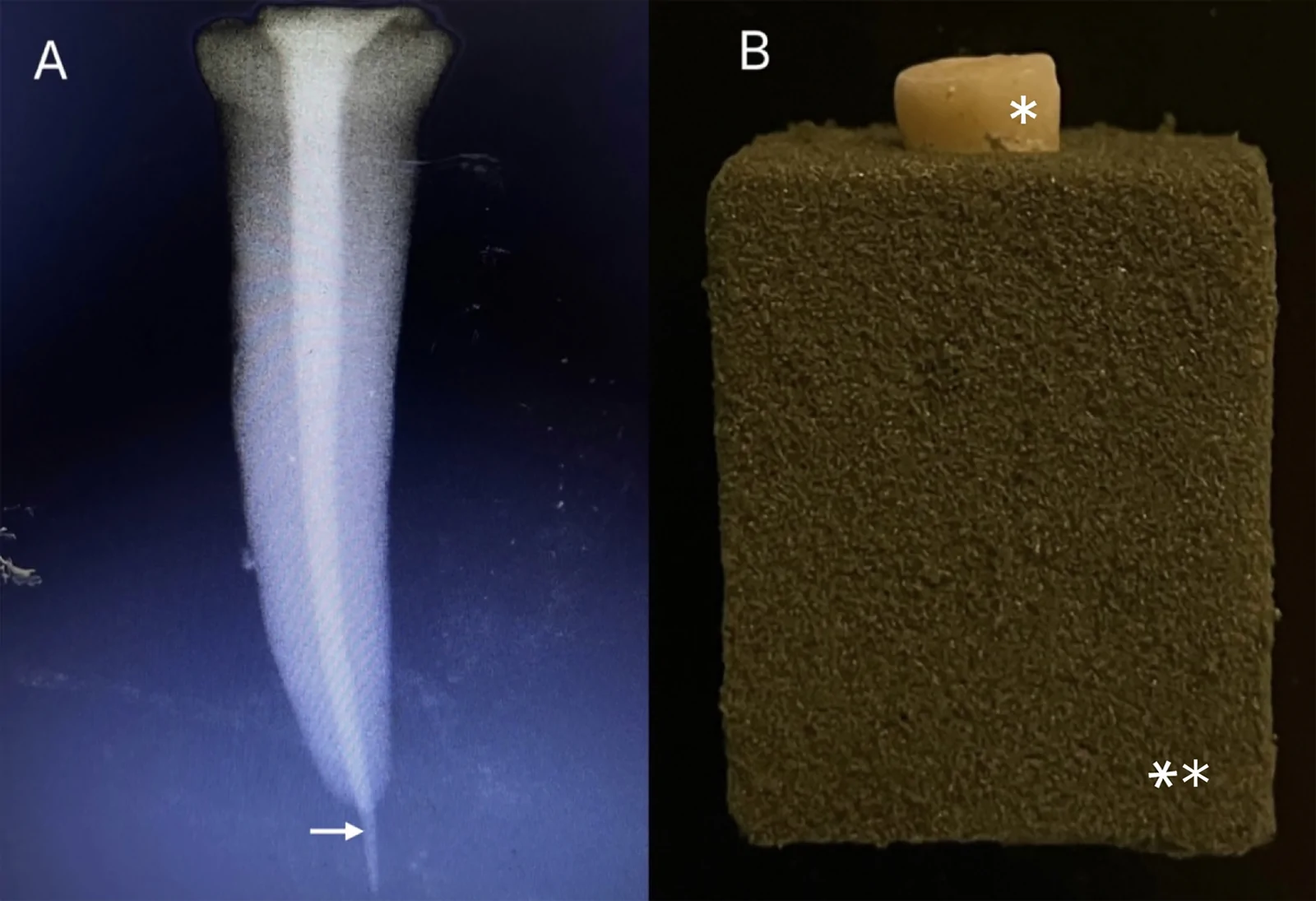
**A** Radiograph of the tooth after standardization and obturation with overextended filling, and (**B**) A tooth embedded in floral foam. (The arrow shows the 3 mm overextended gutta-percha) (* shows the tooth embedded in foam) (** shows the floral foam)

The specimens were inserted into floral foam [15, 16], radiographs were then taken to ensure that the gutta-percha was not bent during embedding. Any specimen with bent gutta-percha was excluded (Fig. 1). The specimens were randomly allocated across three experimental groups (*n* = 12 each).

### Group Ι (H-files)

The coronal portion of the filling material was removed using Gates Glidden drills sizes 3 and 2 (Mani, Japan) at 2000 rpm. Next, H-files (Dentsply Maillefer, Switzerland) of sizes #40, 35, and 30 were used in descending order. Each file was used with circumferential and quarter-turn filing motions to the working length [12] until the overextended root filling was removed.

### Group ΙΙ (PTUR)

PTUR (Dentsply Maillefer, Switzerland) D1, D2, and D3 files were operated respectively using the Endo Gold endomotor (Woodpecker Medical Instrument Co., Ltd., China) at 500 rpm with 3 N⋅cm torque, in brushing motion until complete removal of the overextended filling [12].

### Group ΙΙΙ (XPES + XPEF-R)

Using D-Race (DR1) (30/0.1) (FKG Dentaire SA, La Chaux-de-Fonds, Switzerland) an initial access was created for the subsequent files operating at 800−1000 rpm with 1.5 N⋅cm installed in the endomotor. Next, XPES (30/04) was run at a speed of 800 rpm with 1 N⋅cm, using a pecking motion with light pressure to advance the file for three cycles of 3–4 mm amplitude until the working length. The XPEF-R (30/0) was subsequently run at the same speed and torque using a vertical brushing motion repeated until complete removal of the overextended gutta-percha [12].

**Fig. 2:**
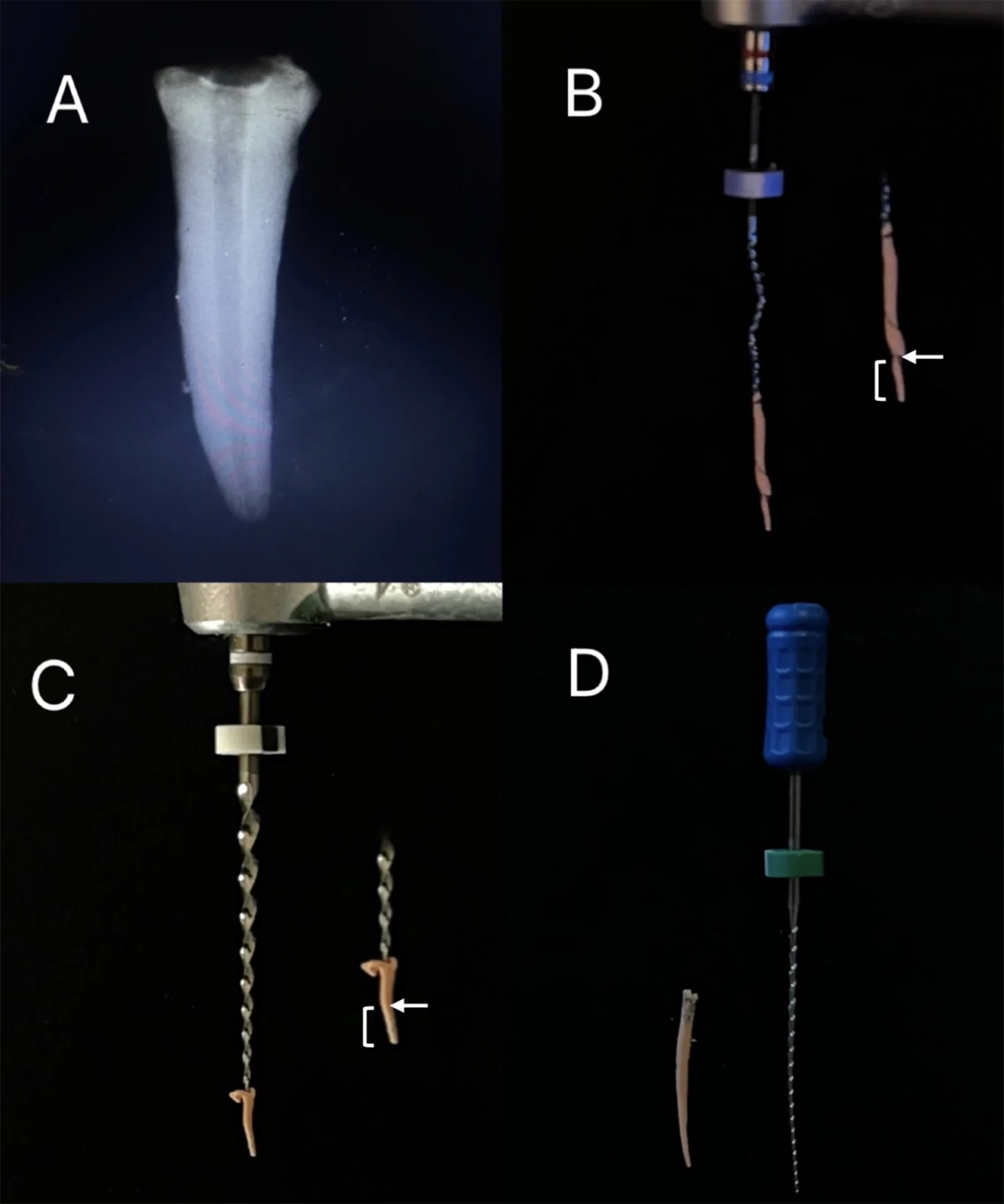
**A** Radiograph of the tooth after complete removal of overextended filling material, and (**B**) The XPER system after removal of the overextended gutta-percha. **C** The PTUR system after removal of the overextended gutta-percha. **D** The H-files system after removal of the overextended gutta-percha. (The arrows showing where the files end). (The square brackets show the 3 mm of gutta-percha which was overextended beyond the apex)

The canal was irrigated with 1 ml of 2.5% NaOCl after each file. The time from the start till complete removal of the overextended filling was recorded and confirmed radiographically (Fig. 2).

### Measuring the time required to remove the overextended root filling

**Fig. 3:**
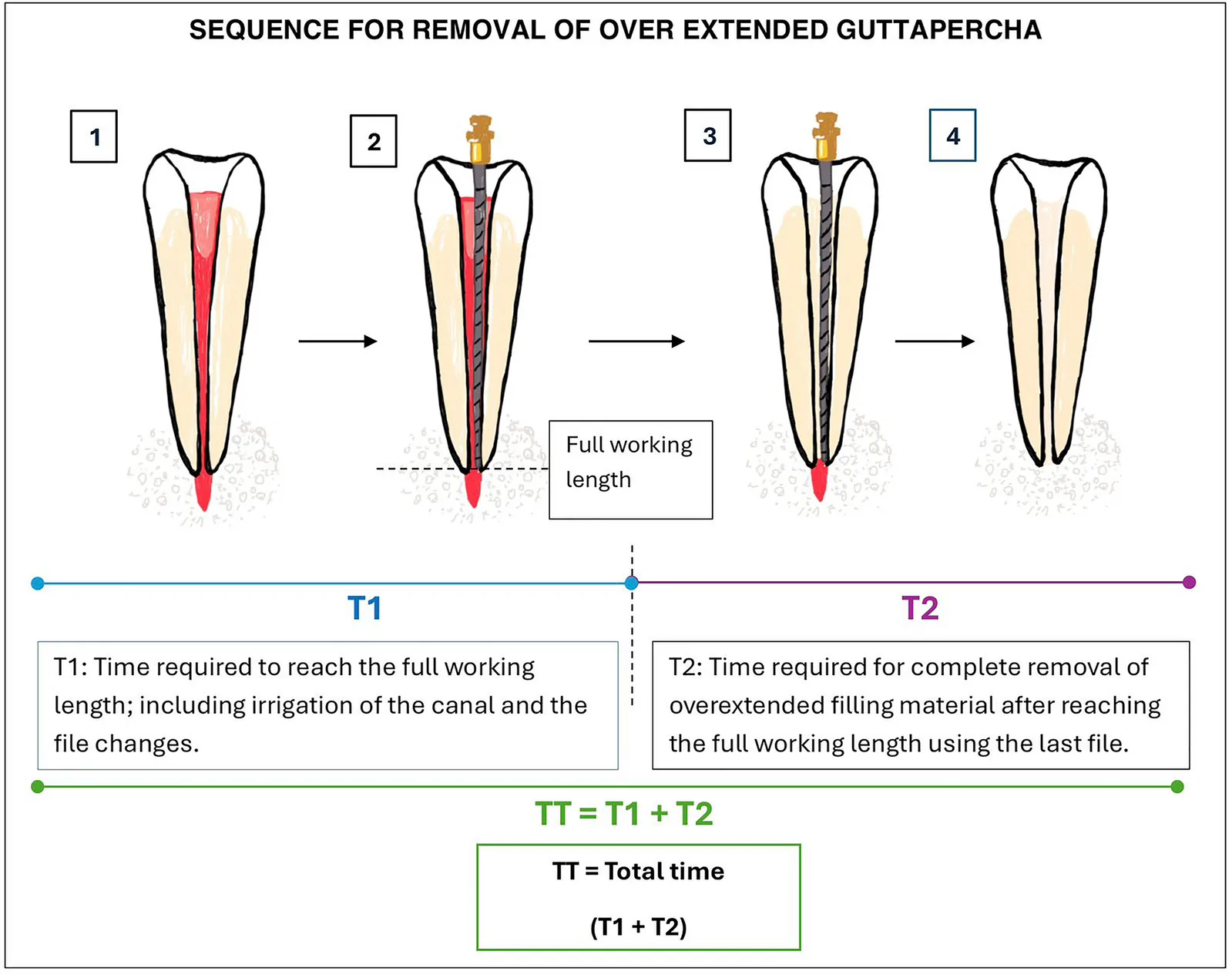
Schematic representation of T1 and T2. (1) Start of retreatment, (2) Progressing through the canal until reaching the full working length, (3) complete removal of the overextended gutta-percha using the last file after reaching the full working length, (4) Complete removal of overextended gutta-percha

For each method, the removal time of overextended obturating material was recorded with a stopwatch. T1 denotes the total time required to reach the full working length, including file changes and irrigation, whereas T2 represents the time required for complete removal of the overextended canal filling after reaching the full working length using the final file. The cumulative time was recorded as TT (Total Time) and calculated as T1 + T2 = TT (Fig. 3) [17].

### Statistical analysis

Normality was assessed using descriptive statistics, Q-Q plots, histograms, and normality tests. All data were normally distributed, therefore parametric tests were employed along with calculations of means and standard deviation (SD). One-way ANOVA was conducted to assess differences among the three study groups, with subsequent multiple pairwise comparisons using Bonferroni correction. T1 and T2 within each group were analyzed for differences using the paired t-test. A p-value of < 0.05 was considered significant. IBM SPSS for Windows (Version 26.0) was used to analyze the data.

## Results

**Fig. 4:**
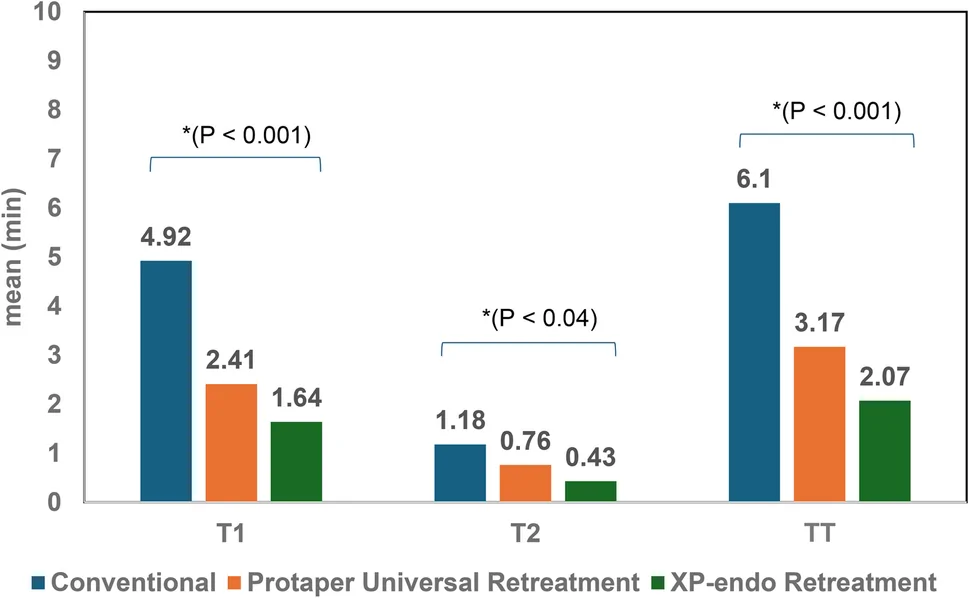
Time in minutes in the three study groups. *Statistically significant at *P* < .05. T1: Time required to reach the working length, including the irrigation of the canal and file changes.T2: Time required for complete removal of filling material after reaching the working length with the last file used for each group. TT: Total time of retreatment (TT = T1 + T2)

During retreatment, the overextended gutta-percha was successfully removed from all specimens. A significant difference was found in the time required for removal among the three groups for T1, T2, and total time (TT) (*P* < .05). The XPER files showed the shortest mean times for T1, T2, and TT, followed by PTUR files, while the H-files required the longest time. These differences were statistically significant for all time points (*P* < .05) (Table 1; Fig. 4). Table 1 also presents a within-group comparison of T1 and T2 using a paired t-test. In all groups, significant differences were observed between T1 and T2 (*P* < .05).

**Table 1:** Comparison of the time required for removal of overextended gutta-percha in minutes between the three study groups (*n* = 12)

		Conventional H-file	Protaper Universal Retreatment	XP-endo Retreatment	*P* value 1
T1	Mean ± SD	4.92 ± 1.10 **a**	2.41 ± 0.20 **b**	1.64 ± 0.13 **c**	< 0.001*
	95% CI	4.22, 5.62	2.28, 2.53	1.55, 1.73	
T2	Mean ± SD	1.18 ± 0.86 **a**	0.76 ± 0.17 **ab**	0.43 ± 0.13 **b**	0.04*
	95% CI	0.63, 1.73	0.65, 0.86	0.35, 0.51	
TT	Mean ± SD	6.10 ± 1.85 **a**	3.17 ± 0.32 **b**	2.07 ± 0.19 **c**	< 0.001*
	95% CI	4.92, 7.28	2.96, 3.37	1.95, 2.19	
*P* value 2 (T1 vs. T2)		< 0.001*	< 0.001*	< 0.001*	

*SD* Standard Deviation

T1: Time required to reach the full working length including the irrigation of the canal and file changes

T2: Time required for complete removal of filling material after reaching the full working length with the last file used for each group

TT: Total time of retreatment (TT = T1 + T2)

*P* value 1: One-way ANOVA comparison between the study groups

*P* value 2: Paired t-test comparison between T1 and T2 within each study group

Different letters (a, b, c) within the same row indicate statistically significant differences between study groups according to Bonferroni-adjusted pairwise comparisons (*P* < .05)

*Statistically significant at *P* < .05

Table 2 This presents a post-hoc comparison of the time (in minutes) required among the three study groups. For T1, a significant difference was found between the H-files group and both the PTUR and XPER groups (*P* < .05), as well as between the PTUR and XPER groups (*P* < .05). For T2, a significant difference was observed only between the H-files group and the XPER group (*P* < .05), while no significant differences were found between the PTUR group and either the H-files or XPER groups (*P* > .05). Finally, for TT, there was a significant difference between the H-files group and both the PTUR and XPER groups (*P* < .05), as well as between the PTUR and XPER groups (*P* < .05).

**Table 2:** Post-hoc comparisons of time in minutes among the three study groups (*n*=12)

	Group	Compared to	*P* value
T1	Conventional H-file	ProTaper Universal Retreatment	<0.001*
		XP-endo Retreatment	<0.001*
	ProTaper Universal Retreatment	XP-endo Retreatment	0.02*
T2	Conventional H-file	ProTaper Universal Retreatment	0.16
		XP-endo Retreatment	0.003*
	ProTaper Universal Retreatment	XP-endo Retreatment	0.39
TT	Conventional H-file	ProTaper Universal Retreatment	<0.001*
		XP-endo Retreatment	<0.001*
	ProTaper Universal Retreatment	XP-endo Retreatment	0.04*

T1: Time required to reach the working length, including the irrigation of the canal and file changes

T2: Time required for complete removal of filling material after reaching the working length with the last file used for each group

TT: Total time of retreatment (TT = T1 + T2)

*Statistically significant at Bonferroni corrected *p*-value

*Statistically significant at *P* < .05

## Discussion

Retreatment involves removing filling material to permit further chemomechanical instrumentation and root canal disinfection [18–22]. Endodontic failure was recognized in teeth with overextended filling material [3–5]. Retreatment success rate can be influenced by tooth type, canal anatomy, and filling removal techniques. Although overextension of obturation material beyond the apical foramen does not invariably lead to treatment failure, it may increase the risk of complications and reduce the overall effectiveness of root canal treatment. Therefore, the root canal obturation should terminate approximately 0.5–1.0 mm short of the anatomical apex [23]. Furthermore, meta-analysis reported higher success rates when obturation terminated 0–1 mm from the apex compared with overextended or > 1 mm short fillings [24]. Ideally, root canal obturation should terminate at the cemento-dentinal junction. However, this anatomical reference point is inconsistent across individuals; typically lying 0–3 mm coronal to the radiographic apex, and may be further influenced by inflammatory processes [25]. In addition, A. ElAyouti et al. found that apical over-instrumentation occurred in 51% of premolar cases, even when the working length appeared appropriate and was set 0–2 mm short of the radiographic apex, leading inadvertently to instrumentation beyond the apex [26]. Minor overextensions may occasionally occur in clinical practice, and greater apical overextension has been associated with a poorer prognosis [3–5, 24]. Apical over-instrumentation and overfilling are undesirable procedural errors and should not be considered acceptable outcomes of root canal treatment. Accordingly, the present study intentionally used a 3-mm extension beyond the apical foramen as a standardized experimental model of pronounced overextension. This model was designed to simulate a particularly challenging retreatment scenario and to provide a standardized condition for comparing the procedural performance and efficacy of the investigated retreatment techniques. It should not be interpreted as suggesting that a 3-mm apical overextension reflects routine clinical practice or constitutes an acceptable treatment endpoint. Rather, this experimental condition was selected solely to enable a rigorous comparison of retreatment protocols in managing severely overextended root canal fillings.

Since the duration required to remove the overextended gutta-percha differed significantly among the studied file systems, the null hypothesis was rejected.

Root canal preparation was performed using the ProTaper Gold rotary system up to the F2 file (25/0.08) (Dentsply Sirona, Switzerland). Although further apical enlargement beyond F2 may enhance canal access and improve debridement, it should be undertaken with caution. Excessive enlargement can result in increased dentin removal, weakening of the root structure, and a potential increase in apical debris extrusion. Therefore, the extent of apical preparation beyond F2 should be determined on an individual case basis, carefully balancing the benefits of improved canal debridement with the need to preserve radicular strength, particularly in anatomically narrow roots such as mandibular premolars.

The root canal sealer type and obturation technique can also influence the residual filling material in the root canal [27]. For standardization, the teeth were trimmed to obtain a uniform tooth length [12], and an epoxy resin-based sealer was used for obturation with cold lateral compaction. Conflicting evidence exists regarding the effect of sealer type on the effectiveness of XPEF-R files. Aksel et al. [28] reported that XPEF file enhanced the elimination of filling material irrespective of the sealer type, while Crozeta et al. [29] reported that the XPEF-R file was effective exclusively with epoxy resin-based sealer.

To mimic intraoral conditions, teeth were embedded in floral foam, which served as a supporting medium capable of partially simulating the surrounding soft tissues and alveolar bone [15, 16]. Although floral foam may provide limited cushioning and support, it cannot fully reproduce the complex biomechanical behavior of the periodontal ligament and alveolar bone. Specifically, it does not accurately replicate the physiological compressive strength, elasticity, and viscoelastic resilience of these tissues. Therefore, while this model may partially approximate in vivo conditions, it remains limited in its ability to fully reflect the clinical situation. Moreover, teeth were stored and retreated at 37 °C, as temperature affects the properties of the XPER files [8].

No studies were reported on the measurement of the time required to remove overextended gutta-percha using XPER files. XPER system demonstrated comprehensive findings in removal of overextended gutta-percha in the least time required with a statistically significant difference (*P* < .05). This stems from the innovative Max wire alloy used to make the files, which combine the shape memory effect with superelasticity [30]. Based on the alloy’s shape-memory property, XPES and XPEF-R expand and contract at body temperature, enabling the files to scrape, contact more walls, and displace filling material [8]. The results are consistent with previous studies [8, 12], which indicated that XPES file, either individually or in combination with XPEF-R file was more effective than other groups.

PTUR system was preferred for this study for its efficiency in gutta percha removal from root canals during retreatment according to Buranade AT et al. [31] PTUR files differ in taper, tip design, file sequence, and type of alloy. The time required by the PTUR files to remove the overextended gutta-percha was greater than that of the XPER group (*P* > .05). This is likely because PTUR files are stiffer, owing to their convex triangular cross-Sect [12].

The slim design of the XPER files with a small taper and an expandable tip enhanced the efficiency of the file in gutta-percha removal [11]. Studies have shown that removing filling material during retreatment is easier when the canal has been enlarged beyond its size prior to obturation [8]. Therefore, the group done using the XPES (30/0.04) and XPEF-R (30/0), which is larger than the initial preparation size F2 (25/0.08), had a significantly shorter mean time than that of the PTUR system group which ended at D3 (20/0.07) (*P* < .05).

H-files group showed the longest time required for removing the overextended gutta-percha (*P* > .05), which is coherent with the investigation undergone by Colaco et al., who asserted that rotary techniques are significantly faster than manual ones (*P* < .05) [32]. Although our results differed from those reported by Atique et al., who found no significant difference in removal time between H-files and ProTaper rotary files (*P* > .05) [33], this discrepancy may be explained by differences in methodology. They removed gutta-percha only along the full length of the canal, whereas in our study, removal was extended beyond the canal length. Furthermore, the current results are consistent with the findings of Orhan et al., who demonstrated that the XPER system exhibited the shortest retreatment time among the evaluated systems [34]. In addition, Alkahtany et al. reported that the XPEF-R file demonstrated potential for effective filling material removal; however, its performance remained inconsistent when compared with other retreatment systems [30]. Moreover, systematic reviews have indicated that XP-endo systems improve cleaning efficacy and retreatment efficiency, while also emphasizing that no currently available technique is capable of completely eliminating residual filling material [20, 35].

Current evidence suggests that all endodontic retreatment techniques result in some degree of apical debris extrusion, which may contribute to postoperative inflammation and influence periapical healing outcomes. A systematic review and meta-analysis has shown that manual instrumentation produces significantly greater apical debris extrusion compared with engine-driven systems, particularly rotary instruments [20, 36]. In contrast, differences between rotary and reciprocating systems are less consistent. Pooled analyses show no statistically significant difference, although some individual studies reporting greater extrusion with reciprocating motion [35].

Various methods have been adopted to evaluate residual gutta-percha after retreatment procedures. However, the present study focused exclusively on the removal of overextended gutta-percha, since further chemomechanical re-instrumentation is usually required after removal of the main bulk of filling material [37]. In this study, ‘complete removal’ was defined as the absence of radiographically detectable overextended gutta-percha on two-dimensional periapical radiographs. We acknowledge that conventional periapical radiography has inherent limitations in detecting residual filling material, especially in anatomically complex or oval canals, and therefore may underestimate the presence of remaining material. Although three-dimensional imaging modalities such as micro-CT provide a more accurate assessment, two-dimensional periapical radiography was intentionally selected to simulate routine clinical conditions and reflect standard diagnostic practice in endodontics. Furthermore, previous studies have also primarily relied on two-dimensional radiographs, supporting the methodology used in the present study. Previous clinical studies, they primarily relied on two-dimensional radiographs when collecting their findings [38, 39]. Furthermore, Baxter et al. reported that flat-panel volume-computed tomography (FpVCT) did not outperform conventional dental radiographs in detecting remaining root-canal-filling material [40]. Consistently, Spinelli et al. stated that both CBCT and X-rays revealed less residual filling material contamination than environmental scanning electron microscopy (ESEM) and energy dispersive X-ray spectroscopy (EDX) [41].

Similarly, beyond imaging considerations, certain methodological constraints also apply to the assessment of procedural outcomes. While both rotary systems demonstrated reduced operating time compared with H-files, the comparison in the present study was based solely on the time required for removal of overextended gutta-percha. Although time efficiency is clinically relevant, it alone cannot determine the overall effectiveness or superiority of a retreatment technique. Nevertheless, procedural time remains a clinically relevant parameter because a shorter removal time reduces the duration of instrumentation and mechanical manipulation required to manage the overextended filling material. This may be particularly relevant when instrumentation is performed in close proximity to or beyond the apical foramen [35]. Reduced manipulation of the apical and periapical regions could theoretically limit tissue irritation; however, this potential biological advantage cannot be inferred directly from the present findings.

Parameters such as the amount of residual filling material, canal cleanliness, apical extrusion of debris, periapical tissue response, postoperative pain, and healing were not evaluated in this study. Previous literature has demonstrated that reduced instrumentation time does not necessarily correlate with superior filling material removal or improved cleaning efficacy during endodontic retreatment [20, 35]. Therefore, the findings of the current study should be interpreted within the context of procedural efficiency only and should not be construed as evidence of overall clinical superiority. Further investigations incorporating qualitative and biological outcome measures are recommended to provide a more comprehensive comparison of retreatment systems. Another limitation of this study is that apical tissues behave differently from floral foam due to foreign body reactions. For instance, a periradicular cyst can form due to chronic inflammation, which may adhere to the overextended gutta-percha, making its removal difficult [42]. Moreover, according to Khabbaz MG, calcified tissue can form around the root apex till the overextended gutta-percha [2]. The management of overextended root canal fillings in lower premolars remains challenging due to their complex root canal anatomy and frequent morphological variations. Evidence suggests that the XPER system demonstrates superior efficiency in gutta-percha removal compared with PTUR system and manual H-files, likely due to its adaptive metallurgy and enhanced canal wall contact. However, variability in canal configuration continues to limit the predictability of any single technique, highlighting the need for individualized approaches. Future research should focus on anatomy-driven retreatment strategies and the integration of adjunctive technologies such as ultrasonic activation or laser-assisted irrigation to improve debridement efficiency and reduce procedural errors. A further limitation is that the study was conducted on teeth with straight or minimally curved roots (< 5°), which does not reflect the clinical reality of retreatment cases where moderate to severe curvatures are common. This may limit the extrapolation of the present findings to complex root canal anatomies.

Further investigations are required to evaluate the effectiveness of the tested files in removing overextended gutta-percha under in vivo conditions, particularly in canals with curvatures exceeding 5°, where outcomes may differ significantly. Moreover, since retreatment efficacy is multifactorial, future studies incorporating volumetric or standardized scoring-based assessments would enhance the clinical relevance of this line of research.

## Conclusions

Regarding this in-vitro study, the XPER system required significantly less time to remove experimentally overextended gutta-percha from mandibular premolars than the PTUR system and H-files. These findings indicate greater procedural time efficiency of the XPER system under the standardized experimental conditions employed in this study. However, shorter removal time should not be interpreted as evidence of overall clinical superiority or improved biological outcomes. Further studies evaluating residual filling material, apical debris extrusion, periapical tissue response, postoperative pain, and healing are required to determine whether the observed reduction in procedural time translates into clinically relevant benefits. In addition, these findings should be interpreted with caution, as the study was limited to straight or minimally curved canals (< 5°) and may not fully represent clinical retreatment scenarios involving curved root canals.

## Data Availability

The datasets used and/or analyzed during the current study are available from the corresponding author upon reasonable request.

## References

[1] Lin LM, Rosenberg PA, Lin J. Do procedural errors cause endodontic treatment failure? J Am Dent Assoc. 2005;136:187–93. 15782522 10.14219/jada.archive.2005.0140

[2] Khabbaz MG, Papadopoulos PD. Deposition of calcified tissue around an overextended gutta-percha cone: case report. Int Endod J. 1999;32:232–5. 10530213 10.1046/j.1365-2591.1999.00209.x

[3] Bergenholtz G, Lekholm U, Milthon R, Engstrom B. Influence of apical overinstrumentation and overfilling on re-treated root canals. J Endod. 1979;5:310–4. 297752 10.1016/S0099-2399(79)80080-1

[4] Swartz DB, Skidmore AE, Griffin JA Jr. Twenty years of endodontic success and failure. J Endod. 1983;9:198–202. 6574207 10.1016/S0099-2399(83)80092-2

[5] Seltzer S. Endodontology: biologic considerations in endodontic procedures. New York: McGraw-Hill Book Company; 1971.

[6] Simões LP, Dos Reis-Prado AH, Bueno CRE, Viana ACD, Duarte MAH, Cintra LTA, et al. Effectiveness and safety of rotary and reciprocating kinematics for retreatment of curved root canals: a systematic review of in vitro studies. Restor Dent Endod. 2022;47:e22. 35692221 10.5395/rde.2022.47.e22 PMC9160764

[7] Bedier MM, Hashem AAR, Hassan YM. Improved dentin disinfection by combining different-geometry rotary nickel-titanium files in preparing root canals. Restor Dent Endod. 2018;43:e46. 30483470 10.5395/rde.2018.43.e46 PMC6237729

[8] Machado AG, Guilherme BPS, Provenzano JC, Marceliano-Alves MF, Gonçalves LS, Siqueira JF Jr, et al. Effects of preparation with the Self-Adjusting File, TRUShape and XP-endo Shaper systems, and a supplementary step with XP-endo Finisher R on filling material removal during retreatment of mandibular molar canals. Int Endod J. 2019;52:709–15. 30417931 10.1111/iej.13039

[9] Uzunoglu-Özyürek E, Küçükkaya Eren S, Karahan S. Contribution of XP-Endo files to the root canal filling removal: A systematic review and meta-analysis of in vitro studies. Aust Endod J. 2021;47:703–14. 33713515 10.1111/aej.12503

[10] Alfadley A, Alrajhi A, Alissa H, Alzeghaibi F, Hamadah L, Alfouzan K, et al. Shaping Ability of XP Endo Shaper File in Curved Root Canal Models. Int J Dent. 2020;2020:4687045. 32148503 10.1155/2020/4687045 PMC7048910

[11] Silva EJNL, Belladonna FG, Zuolo AS, Rodrigues E, Ehrhardt IC, Souza EM, et al. Effectiveness of XP-endo Finisher and XP-endo Finisher R in removing root filling remnants: a micro-CT study. Int Endod J. 2018;51:86–91. 28467618 10.1111/iej.12788

[12] Kapasi K, Kesharani P, Kansara P, Patil D, Kansara T, Sheth S. In vitro comparative evaluation of efficiency of XP-endo shaper, XP-endo finisher, and XP-endo finisher-R files in terms of residual root filling material, preservation of root dentin, and time during retreatment procedures in oval canals - A cone-beam computed tomography analysis. J Conserv Dent. 2020;23:145–51. 33384486 10.4103/JCD.JCD_257_20 PMC7720764

[13] Schneider SW. A comparison of canal preparations in straight and curved root canals. Oral Surg Oral Med Oral Pathol. 1971;32:271–5. 5284110 10.1016/0030-4220(71)90230-1

[14] Mobarak A, Moussa S, Zaazou A, Abdelfattah H. Comparison of bacterial coronal leakage between different obturation materials (an in vitro study). Alex Dent J. 2015;40:1–7.

[15] Lertmalapong P, Jantarat J, Srisatjaluk RL, Komoltri C. Bacterial leakage and marginal adaptation of various bioceramics as apical plug in open apex model. J Investig Clin Dent. 2019;10:e12371. 30468009 10.1111/jicd.12371

[16] Mina MM, Moussa SM, Aboelseoud MR. Marginal adaptation of customized gutta percha cone with calcium silicate based sealer versus MTA and biodentine apical plugs in simulated immature permanent teeth (an in vitro study). BMC Oral Health. 2024;24:1069. 39261838 10.1186/s12903-024-04765-x PMC11391803

[17] Singh KB, Akhtar MS, Nagar R, Agarwal A, Azhar S, Singh V. Comparative analysis of the efficacy of various retreatment file systems in the removal of gutta-percha in retreatment cases and time taken during the procedure: An in vitro cone beam ct study. Cureus. 2024;16:e55551. 38576634 10.7759/cureus.55551 PMC10993640

[18] Schirrmeister JF, Meyer KM, Hermanns P, Altenburger MJ, Wrbas KT. Effectiveness of hand and rotary instrumentation for removing a new synthetic polymer-based root canal obturation material (Epiphany) during retreatment. Int Endod J. 2006;39:150–6. 16454796 10.1111/j.1365-2591.2006.01066.x

[19] Elnagar MK, Mobarak AM, Abdallah AM. Assessment of three heat treated single file systems in gutta-percha removal using cone beam computed tomography (an in vitro study). Alex Dent J. 2023;48:124–31.

[20] Soler-Doria A, Sanz JL, Maddalone M, Forner L. Efficacy of endodontic files in root canal retreatment: a systematic review of in vitro studies. J Funct Biomater. 2025;16:293. 40863313 10.3390/jfb16080293 PMC12387160

[21] Doğan Çankaya T, Işık Aydın M, Uğur Aydın Z. Periapical healing after single-visit non-surgical endodontic retreatment in patients with hypertension: a retrospective study. Sci Rep. 2026;16:5554. 10.1038/s41598-026-35905-8 PMC12890910 41545543

[22] Kara E, Kılıçkesmez A. Retreatment strategies for root canals obturated with calcium silicate-based sealers: A Comprehensive Review. Int J Med Sci Dent Health. 2024;11:1–9.

[23] Wt J. Obturation of the cleaned and shaped root canal system. In: Cohen S, Hargreaves KM, editors. Pathways of the pulp. 9th ed. St. Louis: Mosby Elsevier; 2006. pp. 358–99.

[24] Naito T. Better success rate for root canal therapy when treatment includes obturation short of the apex. Evid Based Dent. 2005;6:45. 16208391 10.1038/sj.ebd.6400335

[25] Gutmann JL, Witherspoon DE. Obturation of the cleaned and shaped root canal system. In: Cohen S, Burns RC, editors. Pathways of the pulp. 8th ed. St. Louis (MO): Mosby; 1989. pp. 293–364.

[26] ElAyouti A, Weiger R, Löst C. Frequency of overinstrumentation with an acceptable radiographic working length. J Endod. 2001;27:49–52. 11487165 10.1097/00004770-200101000-00018

[27] Marchi V, Scheire J, Simon S. Retreatment of Root Canals Filled with BioRoot RCS: An In Vitro Experimental Study. J Endod. 2020;46:858–62. 32354428 10.1016/j.joen.2020.03.018

[28] Aksel H, Küçükkaya Eren S, Askerbeyli Örs S, Serper A, Ocak M, Çelik HH. Micro-CT evaluation of the removal of root fillings using the ProTaper Universal Retreatment system supplemented by the XP-Endo Finisher file. Int Endod J. 2019;52:1070–6. 30715732 10.1111/iej.13094

[29] Crozeta BM, Lopes FC, Menezes Silva R, Silva-Sousa YTC, Moretti LF, Sousa-Neto MD. Retreatability of BC Sealer and AH Plus root canal sealers using new supplementary instrumentation protocol during non-surgical endodontic retreatment. Clin Oral Investig. 2021;25:891–9. 32506324 10.1007/s00784-020-03376-4

[30] Alkahtany SM, Alfadhel R, AlOmair A, Durayhim SB. Characteristics and Effectiveness of XP-Endo Files and Systems: A Narrative Review. Int J Dent. 2024;2024:9412427. 39720541 10.1155/ijod/9412427 PMC11668552

[31] Buranade AT, Algarni YA, Alobaid ASN, Kader MA, Baba SM, Mohamed Ali AB. Comparative Evaluation of Efficacy of Protaper Universal Retreatment System, R-Endo System and Hedstrom File in Gutta Percha Removal During Root Canal Retreatment- an in vitro Study. J Pharm Bioallied Sci. 2022;14:S507–10. 36110617 10.4103/jpbs.jpbs_74_22 PMC9469304

[32] Colaco AS, Pai VA. Comparative Evaluation of the Efficiency of Manual and Rotary Gutta-percha Removal Techniques. J Endod. 2015;41:1871–4. 26364003 10.1016/j.joen.2015.07.012

[33] Atique S, Ali K, Haroon S, Ahmed A, Javed MQ, Zafar MS, et al. Effectiveness of H-files and Pro-Taper universal systems in removing Gutta-percha during endodontic retreatment: a comparative study. J Taibah Univ Med Sci. 2024;19:537–44. 38711796 10.1016/j.jtumed.2024.04.002 PMC11070706

[34] Orhan E, Orhan K, Ocak M, Gunes B. Comparing the root canal filling removal efficiency of XP-endo retreatment system with hand files, R-Endo, Reciproc and ProTaper Universal retreatment systems in curved root canals: a micro-CT study. BMC Oral Health. 2025;25:544. 40217457 10.1186/s12903-025-05849-y PMC11992762

[35] Caviedes-Bucheli J, Rios-Osorio N, Gutiérrez de Pineres-Milazzo C, Jiménez-Peña M, Portigliatti R, Gaviño-Orduña JF, et al. Effectiveness, efficiency, and apical extrusion of 2 rotaries and 2 reciprocating systems in removing filling material during endodontic retreatment. A systematic review. J Clin Exp Dent. 2023;15:e250–63. 37008238 10.4317/jced.59953 PMC10062469

[36] Özyürek EU, Eren SK, Karahan S. Effect of treatment variables on apical extrusion of debris during root canal retreatment: a systematic review and meta-analysis of laboratory studies. J Dent Res Dent Clin Dent Prospects. 2024;18:1. 38881644 10.34172/joddd.40501 PMC11179139

[37] Das S, De Ida A, Das S, Nair V, Saha N, Chattopadhyay S. Comparative evaluation of three different rotary instrumentation systems for removal of gutta-percha from root canal during endodontic retreatment: an in vitro study. J Conserv Dent. 2017;20:311–6. 29386777 10.4103/JCD.JCD_132_17 PMC5767824

[38] Işık M, Aydın ZU. Effect of different obturation techniques on treatment results in single-visit non-surgical endodontic retreatment: randomized controlled clinical study. BMC Oral Health. 2024;24:1449. 39609817 10.1186/s12903-024-05240-3 PMC11603792

[39] Sabeti M, Chung YJ, Aghamohammadi N, Khansari A, Pakzad R, Azarpazhooh A. Outcome of contemporary nonsurgical endodontic retreatment: a systematic review of randomized controlled trials and cohort studies. J Endod. 2024;50:414–33. 38280514 10.1016/j.joen.2024.01.013

[40] Baxter S, Schöler C, Dullin C, Hülsmann M. Sensitivity of conventional radiographs and cone-beam computed tomography in detecting the remaining root-canal filling material. J Oral Sci. 2020;62:271–4. 32493862 10.2334/josnusd.19-0100

[41] Spinelli A, Zamparini F, Buonavoglia A, Pisi P, Gandolfi MG, Prati C. Reciprocating system for secondary root canal treatment of oval canals: CBCT, X-rays for remnant detection and their identification with ESEM and EDX. Appl Sci. 2022;12:11671.

[42] Pai AV, Shrikrishna SB, Shah N. Surgical management of overfilled gutta-percha and root capping with mineral trioxide aggregate in a young patient. J Interdiscip Dent. 2014;4:148–51.

